# Pathogenic variants of meiotic double strand break (DSB) formation genes *PRDM9* and *ANKRD31* in premature ovarian insufficiency

**DOI:** 10.1038/s41436-021-01266-y

**Published:** 2021-07-13

**Authors:** Yiyang Wang, Ting Guo, Hanni Ke, Qian Zhang, Shan Li, Wei Luo, Yingying Qin

**Affiliations:** 1 Center for Reproductive Medicine, Cheeloo College of Medicine, Shandong University, Jinan, Shandong China; 2 National Research Center for Assisted Reproductive Technology and Reproductive Genetics, Shandong University, Jinan, Shandong China; 3 Key laboratory of Reproductive Endocrinology of Ministry of Education, Shandong University, Jinan, Shandong China; 4 Shandong Provincial Clinical Medicine Research Center for Reproductive Health, Shandong University, Jinan, Shandong China

## Abstract

**Purpose:**

The etiology of premature ovarian insufficiency (POI) is heterogeneous, and genetic factors account for 20–25% of the patients. The primordial follicle pool is determined by the meiosis process, which is initiated by programmed DNA double strand breaks (DSB) and homologous recombination. The objective of the study is to explore the role of DSB formation genes in POI pathogenesis.

**Methods:**

Variants in DSB formation genes were analyzed from a database of exome sequencing in 1,030 patients with POI. The pathogenic effects of the potentially causative variants were verified by further functional studies.

**Results:**

Three pathogenic heterozygous variants in *PRDM9* and two in *ANKRD31* were identified in seven patients. Functional studies showed the variants in *PRDM9* impaired its methyltransferase activity, and the *ANKRD31* variations disturbed its interaction with another DSB formation factor REC114 by haploinsufficiency effect, indicating the pathogenic effects of the two genes on ovarian function were dosage dependent.

**Conclusion:**

Our study identified pathogenic variants of *PRDM9* and *ANKRD31* in POI patients, shedding new light on the contribution of meiotic DSB formation genes in ovarian development, further expanding the genetic architecture of POI.

## INTRODUCTION

Premature ovarian insufficiency (POI) is characterized by amenorrhea, infertility, and elevated level of follicle stimulation hormone (FSH > 25 IU/L) in women under the age of 40 [1]. According to the recent meta-analyses of Golezar et al., 3.7% of women are affected by POI [2]. The etiology of POI is heterogeneous, and known causes include genetic, autoimmune, iatrogenic, and infectious factors [3]. Approximately 20–25% of cases have genetic defects [4]. Until now, dozens of genes have been reported to be responsible for POI. However, over 50% of patients are still idiopathic.

Meiosis is initiated by programmed DNA double strand break (DSB) and homologous recombination (HR). Oocytes arresting at the diplotene stage of meiosis I determine the primordial follicles pool and reproductive lifespan of women [5]. In the recent years, with the widespread application of exome sequencing, the spectrum of POI causative genes has been expanded [6]. Interestingly, variants in genes responsible for meiotic HR were enriched in POI etiology, such as *MSH5*, *MCM8*, and *MCM9* [7–9].

Accurate DSB localization and formation are the basis of homologous chromosome recognition, synapsis, and recombination. Most of the DSB formation gene knockout mice, such as *Prdm9*, *Spo11*, *Rec114*, and *Ankrd31*, showed defective meiotic HR, followed by oocytes apoptosis and ovarian dysfunction after birth, which was similar to the phenotype in human POI [10–13]. However, no variant in those genes has been proven causative for POI patients yet. Here, using in-house exome sequencing data in 1,030 patients with sporadic POI, we identified novel variants in *PRDM9* and *ANKRD31*, and the pathogenic effects of variants were further explored.

## MATERIALS AND METHODS

### Participants

The 1,030 POI patients recruited in this exome sequencing project were women with (1) primary or secondary amenorrhea before 40 years of age and (2) at least twice serum follicle-stimulating hormone (FSH) > 25 IU/L at an internal of 4 to 6 weeks. Most of the patients (91.75%) were recruited from Shandong province, China; others were from the north (4.88%) and south (1.86%) of China. Patients with chromosomal abnormalities, ovarian surgery, chemo/radiotherapy, and known autoimmune disease (such as systemic lupus erythematosus, Sjogren syndrome, rheumatoid arthritis, and autoimmune thyroiditis) were excluded. Written informed consents were obtained from all participants.

### Exome sequencing

Peripheral blood was collected from the patients, and the DNeasy Blood & Tissue Kit (Qiagen) was used to isolate the genomic DNA from leukocytes. Exome capture was carried out with SureSelect Target Enrichment System, and sequencing was performed on the Illumina platform (Illumina HiSeq). Reads were aligned against the National Center for Biotechnology Information (NCBI) hg19 reference human genome. Variants were called using ANNOVAR and Genome Analysis Toolkit. The variations in DSB formation genes, including *SPO11*, *PRDM9*, *EWSR1*, *HELLS*, *MEI1*, *MEI4*, *IHO1*, *ANKRD31*, *REC114*, and *TOPOVIBL*, were selected and classified according to American College of Medical Genetics and Genomics/Association for Molecular Pathology (ACMG/AMP) guidelines [14]. The pathogenic or likely pathogenic variations were confirmed by Sanger sequencing.

### Plasmid construction and expression in HEK293 cells

The ANKRD31 functional fragment (NM_001164443.1: 351-1299aa deletion) was synthesized by Changsha Zeqiong Biotechnology Limited Company and cloned into the pcDNA3.1 vector with 3X FLAG tag at the N-terminus. REC114 (NM_001042367) plasmid with HA tag at C-terminus and PRDM9 plasmid (NM_020227.3) with 3X FLAG tag at N-terminus were constructed in the same way. The point variants were generated using the QuikChange Lightning Site-Directed Mutagenesis Kit (Agilent Technologies).

HEK293 cells were cultured in Dulbecco’s Modified Eagle’s Medium (DMEM) with 10% fetal bovine serum and 1% penicillin–streptomycin (Gibco) at 37 °C. The plasmids were transfected into HEK293 cells using Lipofectamine 3000 Transfection Reagent (Invitrogen) according to the manufacturer’s protocol.

### Immunofluorescence microscopy

HEK293 cells were cultured and transiently transfected with wild-type or mutant plasmids for 48 hours. Then, the cells were fixed in 4% paraformaldehyde. After permeabilizing with 0.3% Triton X-100 for 20 minutes and blocking with 10% bovine serum albumin for 1 hour, the cells were incubated with FLAG antibody (rabbit, Cell Signaling) at 4 °C overnight and the secondary antibody conjugated with Alexa Flour 488 (Invitrogen) for 2 hours. DAPI (Beyotime) was used to label DNA. The fluorescent images were captured with the fluorescence microscopes (Olympus, Japan).

### Western blotting

HEK293 cells were transiently transfected with wild-type or mutant PRDM9 plasmids for 48 hours, then cells were harvested using SDS lysis buffer (Beyotime) supplemented with 1% protease inhibitor cocktail (Sigma). Equal amounts of protein were separated on sodium dodecyl-sulfate polyacrylamide gel electrophoresis (SDS-PAGE). Samples were transferred to polyvinylidene fluoride membranes, blocked with 5% nonfat milk diluted in Tris-buffered saline and Tween 20, followed by incubating with antibodies against FLAG (rabbit, Immunoway), H3K4me3 (rabbit, EpiGentek), histone H3 (rabbit, Millipore). After incubating with the secondary antibodies, the blots were subjected to chemiluminescent detection with Chemidoc MP System (Bio-Rad). The grayscale of bands was quantified by Image J software.

### In vitro ovarian culture

To elucidate the effect of *PRDM9* heterozygous loss-of-function variations on follicle survival, we obtained *Prdm9* heterozygous knockout mice from Professor Hongbin Liu at Shandong University [15]. Ovaries were dissected from the wild-type or heterozygous *Prdm9* knockout mice at postnatal 5 days (PD5) and washed three times in Leibovitz’s L-15 medium (Gibco) containing 10% fetal bovine serum and 1% penicillin–streptomycin. Then, the ovaries were transferred into the culture inserts (Millipore) in a 6-well culture plate. The medium containing DMEM/Nutrient Mixture F-12 (DMEM/F12) (Gibco) with 5% insulin–transferrin–selenium (Sigma), 1 mg/mL BSA (Sigma), 1 mg/mL Albumax II (Gibco), 100 µM L-ascorbic (Sigma), and 1% penicillin–streptomycin was used for the in vitro culture. A drop of medium from the well was placed to cover the top of the ovary to prevent drying. Then, the ovaries were cultured at 37°C and 5% CO_2_ for 8 days, and the medium was changed every 2 days. The 4-vinylcyclohexene diepoxide (VCD) (30 µM), which destroys primordial and primary follicles by accelerating the apoptosis of oocytes [16], was used to induce exogenous stress to challenge follicle survival.

### Histology and immunostaining

Ovaries cultured in vitro were fixed in 4% PFA, dehydrated, embedded in paraffin, and sectioned at 5-μm thickness. For Immunostaining, sections were incubated at 4 °C overnight with DDX4 antibody (Goat, Bio Techne) and cleaved PARP antibody (Rabbit, CST), then the sections were incubated with specific secondary antibodies for 1 hour. DAPI was used to mark nucleus. Images were captured with fluorescence microscopes (Olympus, Japan).

### Coimmunoprecipitation (Co-IP)

Plasmids containing ANKRD31 and REC114 were cotransfected into HEK293 cells for 48 hours. Cell protein was extracted in NP-40 lysis buffer (Invitrogen) with 1% protease inhibitor cocktail (Sigma). We adjusted the amount of wild-type and mutant ANKRD31 protein to be equal by bicinchoninic acid (BCA) method. Total of 0.5 mg protein was incubated with 20 μl anti-FLAG M2 magnetic beads (Sigma) at room temperature for 3 hours. Then, the beads were washed twice and boiled with SDS lysis buffer. The supernatant was collected for western blot.

### Minigene assay

The *ANKRD31* c.1565-2A>G variant is located at the donor splice site of intron 10. We acquired the intron 10 (245 bp)–exon 11 (143 bp)–intron 11 (332 bp) fragments by polymerase chain reaction (PCR) and integrated it into pcMINI vector. Two human cell lines (HEK293 and MCF-7cells) were transfected with wild-type or c.1565-2A>G vectors, respectively. After culturing for 48 hours, total RNA was extracted using Trizol (TaKaRa), and complementary DNA (cDNA) was obtained using HifairTM 1^st^ Strand cDNA Synthesis SuperMix (TEASEN).

### Statistical analysis

Software SPSS 21 (IBM) was used for data analysis. The numerical data were compared using independent-samples *t* test and described as mean ± SD. All cytology experiments were repeated at least 3 times; *P* < 0.05 was considered to be statistically significant.

## RESULTS

### Five novel variants of DSB formation genes were identified in POI patients

**Fig. 1:**
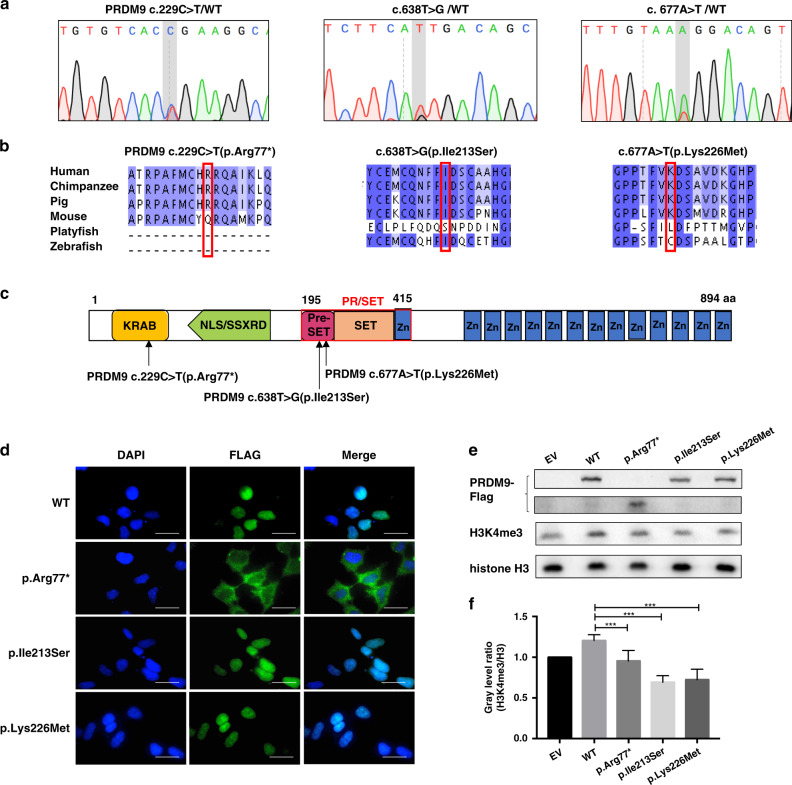
Three pathogenic variants of *PRDM9* identified in premature ovarian insufficiency (POI) patients affected its methyltransferase activity. (**a**) Chromatograms of the three heterozygous variants. (**b**) The mutant amino acids were highly conserved in mammals. (**c**) *PRDM9* c.229C>T (p.Arg77*) localized in the KRAB domain, before the nuclear localization signal (NLS) sequence; both *PRDM9* c.638T>G (p.Ile213Ser) and *PRDM9* c.677A>T (p.Lys226Met) localized on PR/SET domain (residues 195–415), which determined the methyltransferase activity of PRDM9. (**d**) HEK293 cells were transiently transfected with wild-type (WT) or mutant PRDM9 expression vectors, the subcellular location of PRDM9 protein were indicated by FLAG (green). Scale bar: 20 μm. (**e**) H3K4me3 was detected by western blot in HEK293 cells overexpressing empty vector (EV), wild-type (WT) or mutant PRDM9-FLAG. (**f**) The relative grayscale of H3K4me3 was calculated with the use of ImageJ, and compared between subgroups.

**Fig. 2:**
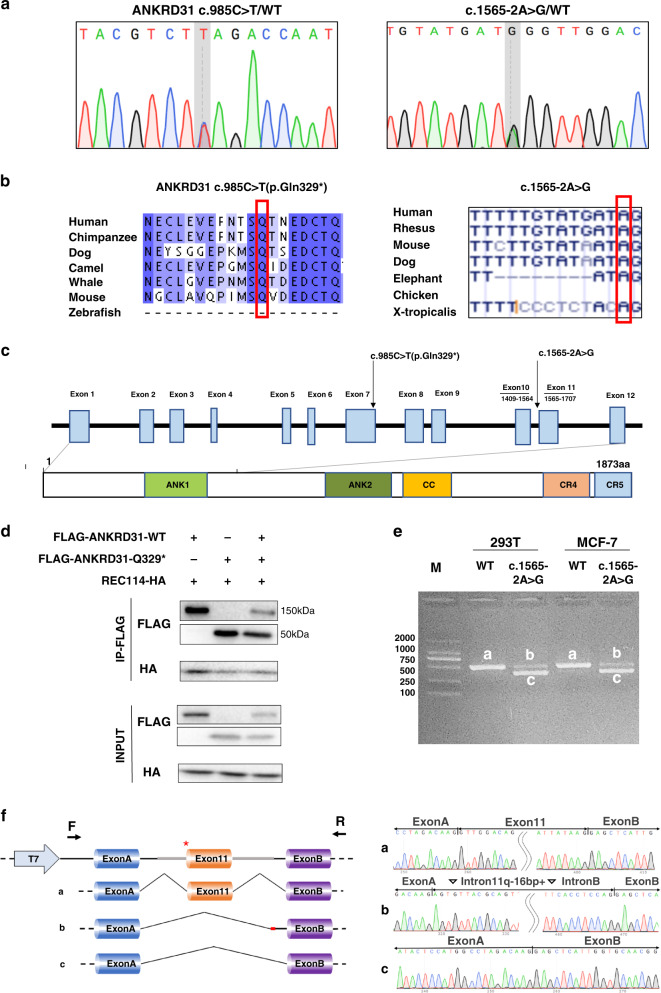
*ANKRD31* p.Gln329* impaired ANKRD31–REC114 interaction and c.1565-2A>G affected RNA splicing. (**a**) Chromatograms of the two heterozygous variants in *ANKRD31*. (**b**) The variants were highly conserved among species. (**c**) *ANKRD31* c.985C>T (p.Gln329*) localized in exon 7, before conserved region 5 (CR5), which was responsible for interaction with REC114. Splice site variant c.1565-2A>G localized at the donor splice site of intron 10. (**d**) Coimmunoprecipitation (Co-IP) analysis showed *ANKRD31* p.Gln329* generated a truncated protein and impaired ANKRD31–REC114 interaction. (**e**) After 48 hours of transfection in two human cell lines (HEK293T and MCF-7 cell), agarose gel electrophoresis showed two belts of c.1565-2A>G transcripts in contrast with wild type. (**f**) Sequence analysis demonstrated that c.1565-2A>G variant caused two transcripts: belt b lacked exon 11 while contained 16 bp of intro11 and intron B; belt c skipped exon 11.

Three heterozygous variants in *PRDM9* (NM_020227.3: c.229C>T, p.Arg77*; c.638T>G, p.Ile213Ser; c.677A>T, p.Lys226Met) and two heterozygous variants in *ANKRD31* (NM_001164443.1: c.1565-2A>G; c.985C>T, p.Gln329*) (ClinVar accessions for the submission are SCV001652780–SCV001652784) were identified in seven POI patients (Table 1, Figs. 1a and 2a). Based on the results of functional studies, they were classified as pathogenic according to ACMG/AMP guidelines. These variant sites were highly conserved among species (Figs. 1b and 2b).

**Table 1:** Pathogenic variants of DSB formation genes identified in POI patients and their clinical characteristics.

Gene name	Gene function	Patient number	Variants identified			Clinical characteristics						
			Genotype	Variant	ACMG/AMP classification	Age at diagnosis, years	Menarche onset, years	Stature, months	BMI	AMH, ng/mL	FSH, mIU/mL	E2,pg/mL
*PRDM9*	Determine the location of DSB hotspots, where genetic recombination occurs	Patient 1	Het	c.229C>T: p.Arg77*	Pathogenic	27	13	1.65	21.3	<0.085	40.64	<5.0
		Patient 2	Het	c.229C>T: p.Arg77*	Pathogenic	23	16	1.58	21.63	0.012	116.5	<5.0
		Patient 3	Het	c.677A>T: p.Lys226Met	Pathogenic	31	15	1.63	27.4	<0.060	99.09	NA
		Patient 4	Het	c.638T>G: p.Ile213Ser	Pathogenic	33	15	1.61	23.15	NA	76.62	60
*ANKRD31*	Act as a scaffold to anchor REC114 and MEI4, thereby regulate DSB formation	Patient 5	Het	c.985C>T: p.Gln329*	Pathogenic	36	16	1.60	25.78	0.98	68.3	<5.0
		Patient 6	Het	c.985C>T: p.Gln329*	Pathogenic	30	14	1.58	24.84	0.002	112.74	22.55
		Patient 7	Het	c.1565-2A>G	Pathogenic	37	13	1.55	31.22	<0.087	45.2	20.7

SI conversion factor: To convert FSH to IU/L, multiply by 1.0; to convert E2 to pmol/L, multiply by 3.671.

*ACMG/AMP* American College of Medical Genetics and Genomics/Association for Molecular Pathology, *AMH* antimullerian hormone, *BMI* body mass index, *DSB* double strand break, *FSH* follicle-stimulating hormone, *Het* heterozygote, *NA* not available, *POI* premature ovarian insufficiency.

PRDM9 is a meiosis-specific histone H3 methyltransferase and a major determinant of meiotic recombination hotspots in mammals. It is consisted of four domains, including the KRAB domain, which promotes protein binding; nuclear localization signal (NLS) sequence; PR/SET domain, which catalyzes trimethylation of histone H3 lysine 4 (H3K4); and the zinc figure sequence, which recognizes the specific DNA motifs of potential hotspots [17]. Variant p.Arg77* was localized in the KRAB domain, and the truncated protein lost the following functional domains. Variants p.Lys226Met and p.Ile213Ser were in the PR/SET domain, which might affect the methyltransferase activity of PRDM9 (Fig. 1c).

ANKRD31 is a recently identified protein regulating DSB formation by interaction with REC114. The nonsense variant p.Gln329* preterminated the protein before the conserved region 5 (CR5), which was responsible for the binding with REC114 [13]. The splice site variant *ANKRD31* c.1565-2A>G before exon 11 (Fig. 2c) was predicted to result in exon skipping and/or intron inclusion, which might result in the loss of functional domain or pretermination of protein.

Variants *PRDM9* p.Arg77* and *ANKRD31* p.Gln329* were carried by two patients separately, and the remaining three variants were found in one patient, respectively. All patients experienced spontaneous menarche and suffered secondary amenorrhea before 38 years old. The clinical characteristics of the heterozygotes are shown in Table 1.

### *PRDM9* p.Arg77* impaired its nuclear localization

To elucidate whether the three variants affected the subcellular localization of PRDM9 protein, wild-type or mutant PRDM9-FLAG plasmids were overexpressed in HEK293 cells. Immunofluorescence against FLAG showed that wild-type PRDM9, mutant p.Lys226Met and p.Ile213Ser were expressed in the nucleus. However, significant reduced nuclear staining of FLAG was observed in cells overexpressing mutant p.Arg77* (Fig. 1d), indicating the truncated protein was resided in cytoplasm due to loss of NLS sequence.

### Three *PRDM9* mutants adversely affected their histone methyltransferase activity

The PRDM9-dependent H3K4 trimethylation (H3K4me3) is essential for determination of recombination hotspots. To illustrate whether the three variants of PRDM9 affected its methyltransferase activity, the level of H3K4me3 was tested among the cells overexpressing wild-type or mutant PRDM9. Results showed that H3K4me3 level was significantly lower in the three mutant groups than in the wild-type group (Fig. 1e, f), indicating the H3K4 methyltransferase activity of PRDM9 was impaired by the three variants.

### *Prdm9*^*+/-*^ oocytes were more vulnerable to exogenous stress

To elucidate the impact of heterozygous loss-of-function variants on oocytes survival, VCD was used to induce exogenous stress. The ovaries were dissected from *Prdm9*^*+/+*^ and *Prdm9*^***+/-***^ mice at PD5 and cultured for 8 days with VCD, and the ovary sections stained with cleaved PARP showed more apoptotic oocytes in *Prdm9*^***+/-***^ ovaries (Fig. S1), indicating the heterozygous knockout oocytes were more susceptible to exogenous stress.

### *ANKRD31* p.Gln329* affected ANKRD31–REC114 interaction

As a meiosis-specific protein, ANKRD31 interacts with REC114 to stabilize and regulate the binding of DSB formation factors (MEI4 and IHO1) onto chromatin [13, 18]. It has been shown that the CR5 region of ANKRD31 directly bonds to the N-terminus of REC114, facilitating the generation of ANKRD31–REC114 heterodimer [13]. Therefore, Co-IP was performed to observe whether the nonsense variant p.Gln329* locating before the CR5 domain impaired the binding with REC114. The result showed that, compared to the wild-type ANKRD31, the truncated protein p.Gln329* had significantly weaker binding with REC114 (Fig. 2d). Furthermore, to illustrate the dominant or haploinsufficiency effect of the heterozygous variant, p.Gln329* and wild-type plasmids were cotransfected into cells with the ratio of 1:1. The level of REC114 pulled down by the cotransfected ANKRD31 was slightly higher than mutant, while lower than wild type, indicating the heterozygous mutant p.Gln329* affected its interaction with REC114 by haploinsufficiency effect.

#### *ANKRD31* c.1565-2A>G resulted in truncated transcripts

To clarify whether the variant c.1565-2A>G disrupted RNA splicing, minigene assay was performed. The pcMINI vector with exon a–intron 10 (245 bp)–exon 11 (143 bp)–intron 11 (332 bp)–exon B (exon A and exon B were the existing exons in pcMINI vector) produced two transcripts both in HEK293 and MCF-7cells: (1) the longer belt (belt b) contained 16 bp of intron 11 and full length of intron B without exon 11; (2) the shorter belt (belt c) lacked exon 11 (Fig. 2e, f). Both two splicing modes would cause frameshift and premature termination, producing two transcripts without the CR5 domain. Therefore, the splice site variant c.1565-2A>G would exert similar pathogenic effect as nonsense variant p.Gln329* with an impaired interaction with REC114.

## DISCUSSION

In the prophase of meiosis I, accurate DSB localization and formation are crucial for homologous chromosome recognition and pairing [19]. Recent studies have found biallelic variants of *MEI1* and *SPO11* in males with familial nonobstructive azoospermia [20, 21], and variants of *TOROVIBL*, *MEI1*, and *REC114* in females with recurrent miscarriage and hydatidiform moles [22, 23]. These studies revealed the essential role of DSB formation genes in human gametogenesis. In the present study, pathogenic variants of *PRDM9* and *ANKRD31* were identified in POI patients, giving the evidence of roles of meiotic DSB formation genes in the maintenance of human follicle pool and ovarian function.

Meiotic DSBs are generated by SPO11 at the hotspots marked by PRDM9-catalyzed H3K4me3 on open chromatin [24]. In *Prdm9*^*-/-*^ mice, DSBs were initiated at PRDM9-independent H3K4me3 sites, such as promoters and enhancers, resulting in aberrant synapsis and recombination that led to germ cell apoptosis [25, 26]. The *Prdm9*^*-/-*^ mice showed accelerated oocyte loss from embryonic day 17.5 and infertility, which was similar to the ovarian phenotype of human POI [10]. In the present study, three novel heterozygous variants of *PRDM9* were identified in four POI patients. Among them, the variant p.Lys226Met and p.Ile213Ser, which localized in the PR/SET domain, adversely affected the methyltransferase activity of PRDM9. Whereas the nonsense variant p.Arg77*, which resulted in the loss of NLS, PR/SET, and zinc finger domain, not only disturbed the nuclear localization of PRDM9, but also suppressed the recognition and methylation of target DNA motifs. Therefore, due to the reduced PRDM9-dependent H3K4me3 sites and thereby defective meiosis HR, women carrying the three variants might experience accelerated oocyte apoptosis. Interestingly, the function of PRDM9 has been proved to be dosage sensitive. *Prdm*9^+/-^ mice were subfertile, and showed increased percentage of germ cells at abnormal pachytene stage with decreased number of PRDM9-dependent DSBs and insufficient recombination [27, 28]. Moreover, in response to VCD induced exogenous stress, the *Prdm*9^+/-^ oocytes demonstrated with increased level of apoptosis, which might explain the reduced number of follicle in *Prdm*9^+/-^ ovaries [29], and the patients carrying heterozygous *PRDM9* variants presented with secondary amenorrhea in this study. Intriguingly, the age of POI onset was much earlier in the two patients carrying variant p.Arg77* (23 years and 27 years, respectively) compared with women carrying p.Lys226Met and p.Ile213Ser (31 years and 33 years, respectively). The former variant lost the NLS and following functional domains of PRDM9, whereas the latter only affected its methyltransferase activity, suggesting the different variation sites might be responsible for the heterogeneous phenotypes of women with POI.

As an essential component of DSB-targeting and control machinery, ANKRD31 interacted with REC114, which stabilized and regulated the localization of DSB formation machinery onto the chromosome axis [13, 18]. ANKRD31 deficiency led to elevated germ cell apoptosis due to altered DSB number, timing, and location. Although *Ankrd3*^*-/-*^ female mice were fertile, they were observed to have 4.95-fold lower median oocyte numbers than that in wild-type mice at 6–7 weeks of age, which lost fecundity much earlier [18]. In our study, two heterozygous variants in *ANKRD31* were identified in three patients. Mutant p.Gln329* generated a preterminated protein lacking CR5 region, which impaired the ANKRD31–REC114 heterodimer formation, while the truncated protein induced by splice site variant c.1565-2A>G also lost the CR5 domain, which would cause dysfunctional DSB formation and meiosis due to the similar effect of variant p.Gln329*. Furthermore, *Ankrd31*^+/-^ mice had increased default DSBs and delayed RAD51 recruitment, suggesting ANKRD31 had a dosage-dependent effect during DSB formation and meiosis [13]. Combined with the observation that the level of REC114 pulled down by cotransfected ANKRD31 (wild-type and mutant) was lower than that pulled down by wild-type protein, the heterozygous variants in *ANKRD31* were suggested to cause POI by the haploinsufficiency effect.

The mechanism that most genes causing ovarian failure by biallelic defects in animal models are heterozygous pathogenic in human has long been discussed. This could be explained partially by a dominant-negative effect, such as the *Nobox* heterozygous variation identified in POI patients [30]. Some other genes presented a dosage-dependent effect on ovarian function both in animal models and patients; for example, heterozygous *Bnc1* and *Fanca* knockout mice showed impaired meiosis or compromised DNA repair [31, 32]. Similarly, the women carrying heterozygous variants were predisposed to POI. Furthermore, some genes without POI phenotypes in heterozygous knockout mice, such as *STAG3*, *MCM8*, and *FANCL*, were found responsible for human POI in heterozygous condition. Haploinsufficiency effect has also been suggested by functional studies [33–36]. The finding that heterozygous pathogenic variants of DNA repair and meiosis genes are related to the POI phenotypes raises the possibility that functional integrity of these genes may be not requisite to maintain ovarian function along the reproductive lifespan but their partial deficiency could lead to POI. In the present study, considering the dosage-dependent effect of *PRDM9* and *ANKRD31* on meiosis and ovarian function in mice, female heterozygotes might be predisposed to POI by haploinsufficiency effect as well.

The follicle pool is determined by the number of primordial germ cells migrating to the genital ridge, followed by germ cell proliferation and functional meiosis, established as the number of primordial follicles at puberty, which will be activated by FSH and recruited to be antral follicles for ovulation [37]. Defects and dysfunction during these processes would cause insufficient follicle formation or accelerated follicle depletion, resulting in POI. The genes involved in follicle activation, development, and steroid hormone synthesis, such as *FIGLA*, *NOBOX*, and *FSHR* [6], were identified to be causative for POI. With the rapid development of next-generation sequencing, the spectrum of POI candidate genes has been greatly expanded. Intriguingly, an increasing number of genes involved in meiotic processes have been found, such as DSB end processing genes *EXO1*, *MND1*, and *MEIOB*; homologous recombination genes *RAD51*, *BRCA2*, *MSH4*, and *MSH5* [38–44]; and synaptonemal complex genes *SYCE1*, *SYCP3*, and *STAG3* [45–47]. The findings in DSB formation genes *PRDM9* and *ANKRD31* further enriched the knowledge of meiotic genes in POI pathogenesis.

In conclusion, we identified pathogenic variants in *PRDM9* and *ANKRD31*; these findings expanded the genetic spectrum of POI, further highlighting the essential role of meiotic DSB formation genes in maintenance of ovarian function.

## Supplementary information

Supplementary information

## Data Availability

The exome sequencing data sets supporting the study have not been deposited in a public repository because of privacy and ethical restrictions but are available from the corresponding authors on request.
