# Supplementary material for: Pathogenic variants of meiotic double strand break (DSB) formation genes PRDM9 and ANKRD31 in premature ovarian insufficiency
Source: Genet Med. 2021 Jul 13;23(12):2309–15. doi: 10.1038/s41436-021-01266-y (PMC8629753; doi:10.1038/s41436-021-01266-y)
Supplement: Supplementary file 1 — Supplementary information [file 41436_2021_1266_MOESM1_ESM.pdf]

Supplementary information

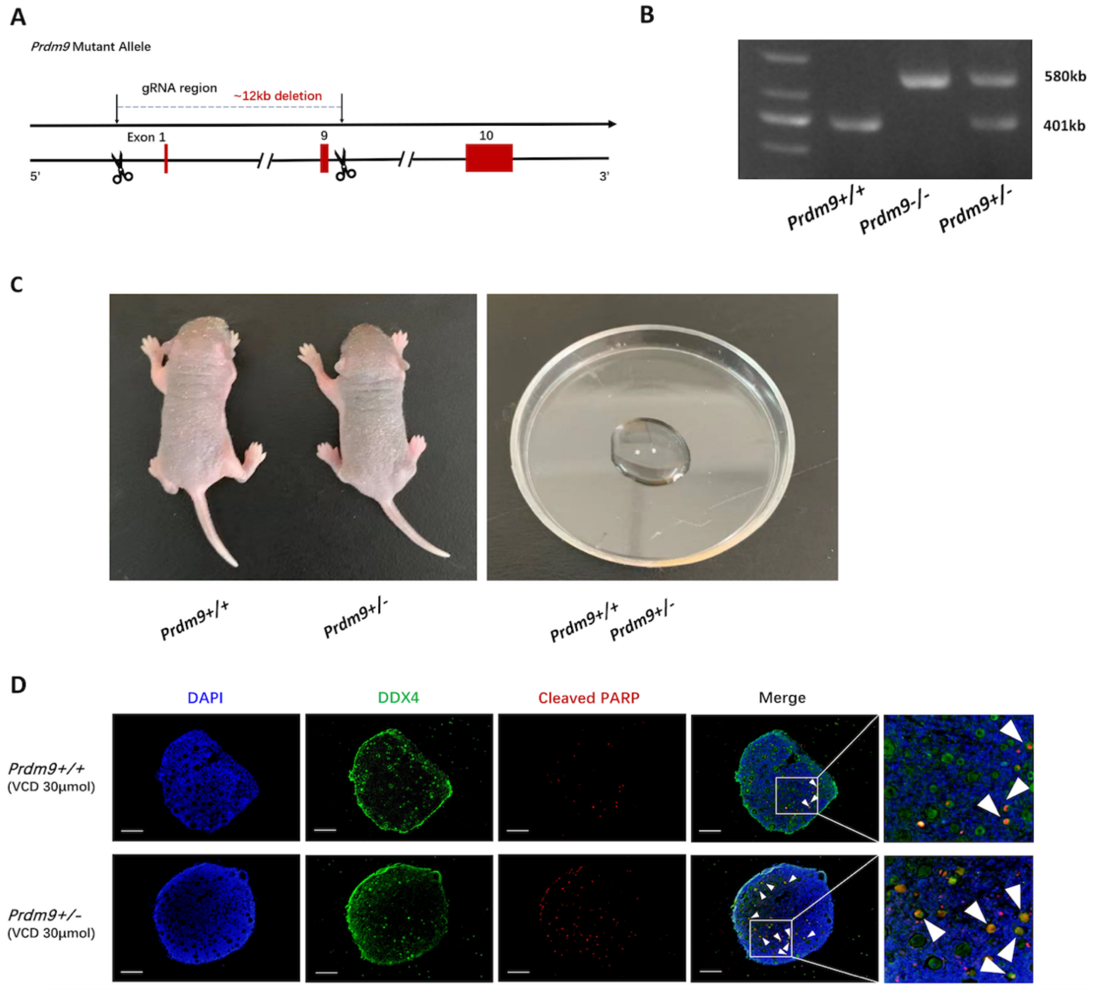

Figure S1. *Prdm9*$^{+/-}$ oocytes were more vulnerable to exogenous stress. (A) Strategy of *Prdm9* knockout mice generation by CRISPR/Cas9. (B) The genotyping results of *Prdm9*$^{+/+}$, *Prdm9*$^{-/-}$ and *Prdm9*$^{+/-}$ mice. (C) *Prdm9*$^{+/+}$ and Prdm9$^{+/-}$ mice at PD5 and the ovaries. Petri dish diameter is 3.5 cm. (D) Sections from cultured ovaries were stained with DAPI (blue, marked DNA), DDX4 (green, marked cytoplasm of oocytes) and Cleaved PARP (red, marked apoptotic cells). The arrows indicated the apoptotic oocytes (DDX4 and Cleaved PARP positive). Scale bar: 100 um.
